# Sleeping to Death

**Published:** 1872-10

**Authors:** 


Sleeping to Death.—The *British Medical Journal,* August 10, 1872,) refers to the singular and always fatal lethargic disease (*lethargus*) occasionally observed to be prevalent among the negroes of West Africa.

Dr. T. H. Bailey, who has observed it there, but who is not able, more than others have been, to explain its pathology, describes it briefly as follows: It is one of the curiosities of medicine. As the name implies, the principal and in fact only symptom that presents itself is lethargy, and one case is essentially a stereotype of all. The patient, usually a male adult, is seized without any premonitory symptom, with a sensation of drowsiness, which continues rapidly to increase, in spite of all efforts to throw it off, until he sinks into a profound and seemingly natural sleep. This continues for about twenty-one days, when death takes place. Throughout the course of the disease, the patient preserves a quiet and peaceful countenance—may be easily aroused for a short time—will take nourishment, and generally answer a few questions in a perfectly rational manner. The pulse, respiration and temperature remain normal throughout; the pupil is neither dilated nor contracted to any noticeable extent, and the urine and fæces are voided with comparative regularity. With the exception of the abnormal tendency to sleep, nothing exists to denote disease." Many careful postmortem examinations have been made by competent men, but nothing of an abnormal character has been found. Dr. Smith, colonial surgeon at Freetown, says that every remedy that could possibly be of any avail had been used, without any apparent beneficial effect. They sleep on and glide into eternity, in spite of professional skill.—*Boston Medical and Surgical Journal.*

Guarana Against Hemicrania.—The guarana, which has already been used for some length of time in France (*Berliner Klin. Wochenschrift,* No. 25, 1872), has lately again been urgently recommended for this disease by Samuel Wilkes, M.D., Physician to Guy's Hospital. It consists of the parched and pulverized seeds of paullinia sorbilis (sapin dacea), and deserves a trial in cases of that kind. R.

